# Perioperative fluid therapy: a statement from the international Fluid Optimization Group

**DOI:** 10.1186/s13741-015-0014-z

**Published:** 2015-04-10

**Authors:** Lais Helena Camacho Navarro, Joshua A Bloomstone, Jose Otavio Costa Auler, Maxime Cannesson, Giorgio Della Rocca, Tong J Gan, Michael Kinsky, Sheldon Magder, Timothy E Miller, Monty Mythen, Azriel Perel, Daniel A Reuter, Michael R Pinsky, George C Kramer

**Affiliations:** Anesthesiology Department, Botucatu Medical School University of Sao Paulo State - UNESP, District of Rubiao Junior s/n, Botucatu, Sao Paulo, 18618-970 Brazil; Valley Anesthesiology Consultants, Ltd., Department of Anesthesia and Perioperative Medicine, Banner Thunderbird Medical Center, Banner Health, Glendale, 85306 AZ USA; Laboratory of Anesthesiology LIM08, Medical School - University of São Paulo, São Paulo, 05508-070 São Paulo Brazil; Department of Anesthesiology & Perioperative Care, University of California, Irvine, 92697 CA USA; Department of Anesthesia and ICM, University of Udine, Udine, 33100 Italy; Department of Anesthesiology, Duke University Medical School, Durham, 27710 NC USA; Resuscitation Research Laboratory, Department of Anesthesiology, University of Texas Medical Branch, Galveston, 7755-0801 TX USA; Medicine and Physiology, McGill University, Montreal, H3A 0G4 QC Canada; University College London Hospital, 235 Euston Road, Fitzrovia, London, NW1 2BU UK; Department of Anesthesiology and Intensive Care, Sheba Medical Center, Tel Aviv University, Aviv, 52621 Israel; Center of Anesthesiology and Intensive Care Medicine, Hamburg Eppendorf University Medical Center, Hamburg, 20246 Germany; Department of Critical Care Medicine, University of Pittsburgh, Pittsburgh, 15213 PA USA

**Keywords:** Fluid resuscitation, Perioperative fluids, Goal-directed fluid therapy, Fluid responsiveness

## Abstract

**Background:**

Perioperative fluid therapy remains a highly debated topic. Its purpose is to maintain or restore effective circulating blood volume during the immediate perioperative period. Maintaining effective circulating blood volume and pressure are key components of assuring adequate organ perfusion while avoiding the risks associated with either organ hypo- or hyperperfusion. Relative to perioperative fluid therapy, three inescapable conclusions exist: overhydration is bad, underhydration is bad, and what we assume about the fluid status of our patients may be incorrect. There is wide variability of practice, both between individuals and institutions. The aims of this paper are to clearly define the risks and benefits of fluid choices within the perioperative space, to describe current evidence-based methodologies for their administration, and ultimately to reduce the variability with which perioperative fluids are administered.

**Methods:**

Based on the abovementioned acknowledgements, a group of 72 researchers, well known within the field of fluid resuscitation, were invited, via email, to attend a meeting that was held in Chicago in 2011 to discuss perioperative fluid therapy. From the 72 invitees, 14 researchers representing 7 countries attended, and thus, the international Fluid Optimization Group (FOG) came into existence. These researches, working collaboratively, have reviewed the data from 162 different fluid resuscitation papers including both operative and intensive care unit populations. This manuscript is the result of 3 years of evidence-based, discussions, analysis, and synthesis of the currently known risks and benefits of individual fluids and the best methods for administering them.

**Results:**

The results of this review paper provide an overview of the components of an effective perioperative fluid administration plan and address both the physiologic principles and outcomes of fluid administration.

**Conclusions:**

We recommend that both perioperative fluid choice and therapy be individualized. Patients should receive fluid therapy guided by predefined physiologic targets. Specifically, fluids should be administered when patients require augmentation of their perfusion and are also volume responsive. This paper provides a general approach to fluid therapy and practical recommendations.

## Background

### Fluid therapy is important

Major surgery is a considerable physiologic insult that can be associated with significant morbidity and mortality. The occurrence of one or more postoperative complications adversely effects both short-term and long-term survival and increases healthcare costs [1,2]. The prevention of postoperative morbidity is a key factor in providing high-quality, high-value health care.

Perioperative fluid management remains a highly debated topic. There is wide variability of practice, both between individuals and institutions. Perioperative morbidity is linked to the amount of intravenous fluid administered (fluid therapy) with both insufficient and, more commonly, excess fluid delivery leading to increased postoperative complications [3-5]. Currently taught and practiced methods of intraoperative volume management in which intravenous fluids are given based on a generalizable formula relying on body weight per unit time and modified by the perceived magnitude of surgical ‘trauma’ [6] are not supported by known physiologic principles. Fluid therapy should be considered when patients are both in need of enhanced blood flow and are fluid responsive.

Multiple studies have shown that approaching fluid therapy with the goal of hemodynamic stabilization can reduce complications after major surgery [7-9]. More compelling are several meta-analyses and quantitative reviews demonstrating the strength of these beneficial effects across patient groups and surgical procedures [8,10]. It is the purpose of this review to provide an overview of the components of an effective perioperative fluid administration plan.

### The physiologic principles of fluid support

A patient’s physiologic status in general and hemodynamic stability in particular define the need for cardiovascular support, including fluid therapy and use of vasoactive drugs (vasopressors, vasodilators) and inotropes. Specific hemodynamic goals include maintaining adequate blood volume and sustaining perfusion pressure so as to maintain cardiac output, tissue blood flow, and adequate oxygen delivery. Fluid therapy is often the first line of hemodynamic support because decreased effective circulating blood volume often accompanies induction of anesthesia and surgical trauma. However, fluid therapy only indirectly impacts cardiac and vascular function. Optimizing oxygen delivery and assuring the removal of metabolic bioproducts may require a combination of individualized fluid therapy, pharmacotherapy, and occasionally mechanical cardiovascular support.

**Figure 1:**
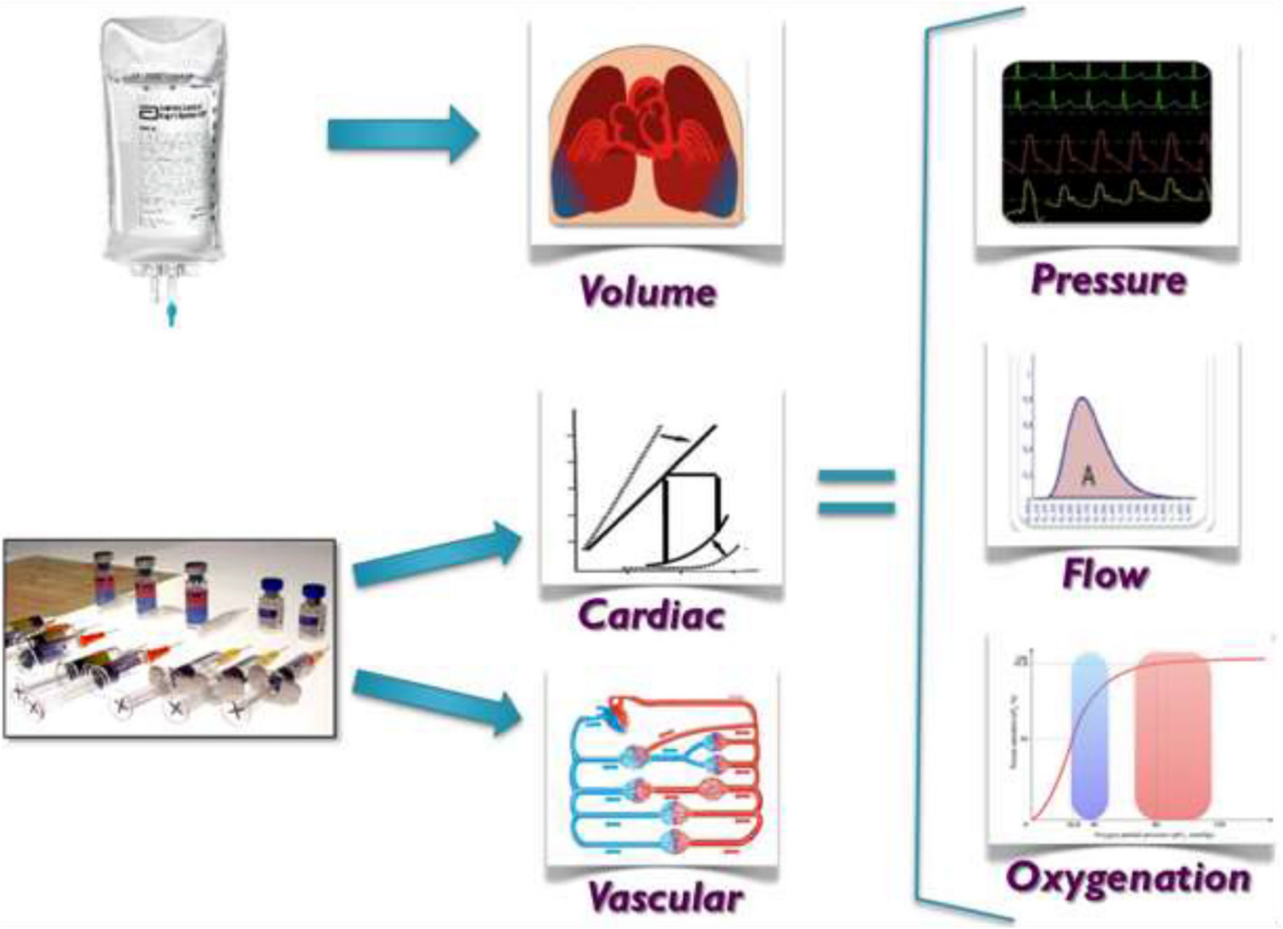
**Perspective of the anesthesiologist’s tools (fluid and drugs) and the physiologic targets of these tools (blood volume, the heart, and blood vessels).** The heart has two components (contractility and rate), and the blood vessels have two major characteristics (compliance and resistance). It is blood volume, heart, and blood vessels that produce pressure, flow, and oxygen delivery, while the intermediate physiologic functions and their metrics provide a means of assessing the cardiovascular state and how effective fluids are likely to be.

Fluid infusions directly increase vascular volume, subsequently and usually improve global and regional perfusion and blood pressures if the heart is preload-responsive, and often improve oxygen delivery and tissue oxygenation. However, these changes are profoundly influenced by the cardiac and peripheral vascular status [11]. Thus, the same fluid therapy can have profoundly different and occasionally opposite changes in cardiovascular state. For this reason, the blind infusion of fluids or the use of vasopressors without first understanding the patient’s cardiovascular reserve is discouraged. Given these physiologic principles, hemodynamic optimization requires that the anesthesiologist consider three specific therapies for each patient: 1) fluid therapy for the correction of volume deficits associated with insufficient circulating blood volume and oxygen delivery, 2) vasopressors and vasodilators for arterial pressure and vascular tone, and 3) inotropic support when cardiac output remains inadequate despite optimization of volume (Figure 1).

Intravenous infusion of fluid directly expands plasma volume with transient or sustained effect that varies based on the colloid osmotic properties of the fluid, blood flow distribution, type and level of anesthesia, vascular endothelial integrity, and the physiologic state. The expansion of plasma volume causes the mean systemic pressure to increase, and, if greater than right atrial pressure, the pressure gradient for venous return will increase. If the right and left ventricle are volume responsive, then cardiac output will also increase. There is no easy means to measure plasma volume nor is there a defined means of how measured plasma volume could be used to achieve the physiologic goals of optimal pressure, flow, and oxygenation for the perioperative patient. Although one can estimate mean systemic filling pressure in appropriately instrumented patients, it is unclear if such measures will alter either therapy or outcome, because knowing plasma volume and even effective circulating blood volume only gives a partial picture of the determinants of cardiac output. Other critical factors include blood flow distribution, vasomotor tone, right ventricular function, and the level of positive-end expiratory pressure, which, individually and collectively, may alter cardiovascular responsiveness. Given the absence of easily obtainable regional measures of perfusion, the anesthesiologist may consider assessing global perfusion by measuring base deficit, lactate, and central and mixed venous oxygen saturation to clarify the impact of selected interventions.

Perioperative assessment of changes in blood volume is difficult and requires evaluation of several clinical and physiologic events that accompany major surgery. Standard hemodynamic monitoring devices fail to detect occult hypovolemia [12], which occurs frequently during surgery and contributes to inadequate tissue perfusion and the development of postoperative complications. Severely compromised patients may be identified by the presence of hypotension; however, not all patients in shock are hypotensive, and if one waits for hypotension, tissue hypoperfusion has already occurred [13]. For example, studies in healthy volunteers have shown that blood volume losses of 20% to 30% may occur with minimal change in blood pressure despite measurable impairment of tissue perfusion [14]. Moreover, hypotension should not serve as an automatic trigger for fluid administration since not all hypotensive events are due to hypovolemia.

Tachycardia is considered a classic sign of hypovolemia, but the assessment of intravascular volume based on heart rate lacks sensitivity and specificity [15] for a variety of reasons, not minimally because of the common use of beta-adrenergic receptor blocking agents in older surgical patients.

Perioperative hypovolemia is deleterious to organ function because normal adaptive mechanisms cause peripheral vasoconstriction to sustain blood flow to the heart and brain, causing ischemia to other organ and surgical tissues in need of blood flow for repair. For surgical patients, several factors including preoperative fasting, hypertonic bowel preparations, anesthetic agents, and positive pressure ventilation all contribute to reduced effective circulating blood volume. Anesthetized patients often present with a functional intravascular volume deficit [7]. On the other hand, large volumes of intravenous fluid may cause complications due to the formation of tissue edema. Liberal administration of fluid may impair pulmonary, cardiac, gastrointestinal, and renal function, contributing to postoperative complications and prolonged recovery [5,16-20].

Establishing what constitutes a restrictive or liberal amount of fluid from the literature is difficult because the absolute amounts of fluid administered vary substantially among trials making any conclusion difficult to implement in clinical practice [21]. Several studies have shown that the absolute amount of perioperative fluid administered may not be a major determinant of perioperative outcomes. Titration of fluid according to a hemodynamic goal is pivotal in improving perioperative outcomes [22]. In some studies, improved outcomes have been reported when set guidelines of ‘restrictive’ or ‘limited’ fluid therapy have been compared to standard care for GI surgeries [23-25] and in patients with pulmonary dysfunction [26,27]. These studies would seem to speak against individualized goal-directed therapy that is predicated upon optimizing intravascular volume. However, most certainly, the restrictive fluid studies and the goal-directed therapy (GDT) trials both make a strong case for having an *a priori* perioperative fluid plan. Taken as a whole, the success of both GDT and of some restrictive fluid strategies suggests that perioperative fluid planning must emphasize that fluid therapy be administered only with clear indication. Functional hemodynamic parameters offer unique information about fluid responsiveness, which my help detect fluid needs and avoid unnecessary fluid loading. Despite their limitations and confounding factors, this information may be crucial in guiding fluid therapy in surgical patients [28]. The exact set points and target values for the restoration and optimization of circulating volume, pressure, and perfusion must be determined for each patient.

### Perioperative goal-directed therapy impacts clinical outcomes

Individual clinical trials and meta-analyses have shown that different fluid therapy regimens produce significantly different clinical outcomes and have resulted in considerable controversy as to the best approach. Table 1 lists trials of GDT trials applied within the perioperative space [23,29-59]. Most of these studies report higher rates of complications within the control groups. In high-risk surgical patients, perioperative fluid overload is associated with life-threatening complications, including pulmonary edema and death [60,61]. Interestingly, the application of specific GDT protocols has often been associated with increased delivery of fluids, especially colloids (Table 1), and in some studies less. Taken together, these data suggest that the benefit of fluid therapy is not primarily related to the volume infused, but rather how and when volume therapy is administered to a given patient.

**Table 1:** **Trials of goal-directed therapy** [23,29-59]

**Protocols**				**Fluids GDT** ***versus*** **control**			
**Population**	**GDT endpoints**	**GDT therapy**	**Control protocol**	**Crystalloids**	**Colloids**	**Outcomes GDT** ***versus*** **control**	**Reference**
Elective cardiac surgery	ΔSV < 10% (esophageal Doppler)	Bolus 200 ml colloid	Standard of care	Less	More	Reduction of gut mucosal hypoperfusion, less postoperative complications, shorter ICU stay, shorter HLOS	Mythen and Webb [29]
	ΔCVP < 3 mmHg						
Proximal femoral fracture repair	FTc > 400 ms, ΔSV < 10% (esophageal Doppler)	Bolus 3 ml/kg colloid	Standard of care	Similar	More	Shorter HLOS	Sinclair *et al*. [30]
Transthoracic esophagectomy	CVP < 5 mmHg	Restrictive regimen	Standard of care	No data	No data	Less postoperative pulmonary complications	Kita *et al*. [31]
Major bowel surgery	FTc > 350 ms	Bolus 3 ml/kg colloid	Standard of care	No data	More	Less critical care admission	Conway *et al*. [32]
	ΔSV < 10% (Doppler)						
Major elective surgery	FTc > 350 ms	Bolus 200 ml colloid	Standard of care (HR, CVP, MAP, UO)	Similar	More	Less PONV, earlier oral solid intake, shorter HLOS	Gan *et al*. [33]
	ΔSV < 10% (Doppler)						
Proximal femoral fracture repair	Doppler - FTc > 400 ms,	Bolus 200 ml colloid	Standard of care (without CVP or Doppler)	Similar	More	Less intraoperative hypotension, sooner medically fit for discharge	Venn *et al*. [34]
	ΔSV < 10%						
	CVP - ΔCVP < 5 mmHg						
Elective colorectal resection	Maintaining preoperative body weight	Restrictive regimen	Standard of care	Less	Similar	Less postoperative complications (tissue healing, cardiopulmonary)	Brandstrup *et al*. [35]
High-risk surgical patients (≥60 years old)	DO_2_ = 550 to 600 ml/min/m^2^	Fluids, inotropes, vasodilators, vasopressors, RBC	Standard of care (without PAC)	No data	No data	More pulmonary embolism	Sandham *et al*. [36]
	CI = 3.5 to 4.5 l/min/m^2^						
	MAP = 70 mmHg						
	HR < 120 bpm, Ht ≥ 27%						
Colorectal resection	ΔSV < 10% (Doppler)	Bolus 250 ml colloid	Routine monitoring (CVP = 12 to 15 mmHg)	Similar	More	Shorter recovery of gut function, less morbidity, shorter HLOS	Wakeling *et al*. [37]
	ΔCVP < 3 mmHg						
Elective colorectal resection	FTc > 350 ms	7 ml/kg first bolus colloid, then bolus 3 ml/kg colloid	Standard of care (without bolus)	Similar	Similar	Less inotrope use, earlier diet, less days to medically fit, shorter HLOS	Noblett *et al*. [38]
	ΔSV < 10% (Doppler)						
Low-risk patients off-pump coronary surgery	PAC	No data	Standard of care (CVP)	No data	No data	More use of inotropes	Resano *et al*. [39]
Major abdominal surgery	O_2_ER < 27%	Colloid bolus, RBC, dobutamine	Standard of care (MAP, UO)	No data	No data	Less organ failure, shorter HLOS	Donati *et al*. [40]
Cardiac bypass surgery	GEDVI = 640 ml/m^2^	Bolus 500 ml, vasopressors	Standard of care (CVP, MAP, clinical evaluation)	Similar	More	Shorter and reduced need for vasopressors, mechanical ventilation, and ICU therapy	Goepfert *et al*. [41]
	CI > 2.5 l/min/m^2^						
	MAP = 70 mmHg						
High-risk surgery	ΔPP < 10%	Bolus colloid	Standard of care	Similar	More	Less postoperative complications, shorter time of mechanical ventilation, ICU stay and HLOS	Lopes *et al*. [42]
Moderate to high-risk cardiac surgery	DO_2_ = 450 to 600 ml/min/m^2^	Bolus 100 ml colloid	CVP = 6 to 8 mmHg	Similar	More	Lower number of adjustments of inotropic agents	Kapoor *et al*. [43]
	CI = 2.5 to 4.2 l/min/m^2^		MAP = 90 to 105 mmHg				
	SVI = 30 to 65 ml/beat/m^2^		UO > 1 ml/kg/h				
	ScvO_2_ > 70%, SVV < 10%						
Off-pump coronary surgery	ITBVI > 850 ml/m^2^	Bolus 500 ml colloid	Standard of care (MAP, CVP, HR)	Similar	More	Shorter HLOS	Smetkin *et al*. [44]
	ScvO_2_ > 60%						
Laparoscopic segmental colectomy	2 GDT groups:	Bolus 200 ml colloid or 300 ml crystalloid	Standard of care	More (GDT crystalloid)	More (GDT colloid)	More postoperative complications on group GDT colloid	Senagore *et al*. [45]
	ΔSV < 10%						
	Crystalloids *versus* colloids						
Major abdominal surgery	PVI < 13%	Bolus 250 ml colloid (norepinephrine to MAP > 65 mmHg)	Standard of care (MAP, CVP)	Less	Similar	Lower lactate levels	Forget *et al*. [46]
Elective surgery for GI malignancy	Serum lactate < 1.6 mmol/l	Bolus 250 to 1,000 ml colloid (depending serum lactate)	Restrictive regimen	Similar	Similar	Less systemic complications in patients that need postoperative supplementary fluids	Wenkui *et al*., [47]
Major abdominal surgery	Peak aortic flow velocity < 13% (Doppler)	Bolus 250 ml, vasopressors, dobutamine, restrictive crystalloids	Standard of care (12 ml/kg/h crystalloids)	Less (patients with complication)	More (patients with complication)	More postoperative complications	Futier *et al*. [48]
Peripheral artery bypass grafting	CI > 2.5 l/min/m	Bolus 250 ml colloid, dobutamine	Standard of care (MAP, CVP)	No data	Similar	No difference between groups	Van der Linden *et al*. [49]
Major abdominal surgery	CI > 2.5 l/min/m^2^	Bolus 500 ml crystalloid, bolus 250 ml colloid, dobutamine, norepinephrine	Standard of care (MAP, CVP, UO)	Less	More	Less postoperative complications, shorter HLOS	Mayer *et al*. [50]
	SVI > 35 ml/beat/m^2^						
	MAP > 65 mmHg						
Elective intra-abdominal surgery in high-risk patients	SVV < 10%	Bolus 3 ml/kg colloid, dobutamine	Standard of care (MAP > 65 mmHg, HR < 100 bpm, CVP = 8 to 15 mmHg, UO > 0.5 ml/kg/h)	Similar	More	Better intraoperative hemodynamic stability, lower serum lactate, less postoperative complications	Benes *et al*. [51]
	CI > 2.5 l/min/m^2^						
Elective total hip replacement	DO_2_ > 600 ml/min/m^2^	Bolus 250 ml colloid, dobutamine, RBC	Standard of care (MAP)	More	More	Less postoperative complications, (hypotension, cardiovascular)	Cecconi *et al*. [52]
	ΔSV < 10%, Hb > 10 g/dl						
Elective colorectal surgery	ΔSV < 10%	Bolus 200 ml colloid	Zero balance intraoperative fluids (MAP > 60 mmHg)	Similar	More	No difference between groups	Brandstrup *et al*. [23]
Major abdominal surgery (cirrhotic patients)	2 GDT groups:	Bolus 250 ml LR followed by 3 ml/kg colloid	Same for both groups	Similar	Similar	No difference between groups	Abdullah *et al*. [53]
	PVI < 13%						
	FTc > 350 ms						
Major colorectal surgery	ΔSV < 10%	Bolus 200 ml colloid	Standard of care	Similar	More	More blood loss and need for transfusion in OR, longer HLOS	Challand *et al*. [54]
Noncardiac major surgery	FTc > 300 ms, ΔSV < 10%	Bolus 200 ml colloid	Bolus 200 ml crystalloid	Less	More	Less transfusion of FFP, better hemodynamic stability	Feldheiser *et al*. [55]
	MAP > 70 mmHg						
	CI > 2.5 l/min/m^2^						
Elective colectomy	FTc > 400 ms	7 ml/kg first bolus colloid, then bolus 3 ml/kg colloid	Restrictive regimen	Similar	More	No differences in outcomes	Srinivasa *et al*. [56]
	ΔSV < 10%		(HR, MAP, UO)				
Cytoreductive surgery (ovarian cancer)	ΔSV < 10%	Bolus 200 ml	200 ml crystalloid	Less	More	Better hemodynamic stability, less FFP transfusion	Feldheiser *et al*. [57]
Major abdominal surgery	CI > 2.5 l/min/m^2^	Fluids, dobutamine, vasopressors	Standard of care	Similar	Similar	Less postoperative complications, lower infection rate	Salzwedel *et al*. [58]
	PPV < 10%						
	MAP > 65 mmHg						
Major abdominal surgery	CO SV	Bolus 250 ml colloid	Standard of care (CVP)	Less	More	No difference in outcomes	Pearse *et al.* [59]

Individual clinical trials and meta-analyses have shown that different fluid therapy regimens produce significantly different clinical outcomes and have resulted in considerable controversy as to the best approach. This table represents a summary of the known peer-reviewed GDT trials including their physiologic targets, fluids used, and outcomes measured.

The use of standardized fluid therapy protocols within the perioperative space is limited despite strong evidence of benefit. A survey by Cannesson *et al*. compared the fluid therapy practices of both American Society of Anesthesiologists (ASA) and European Society of Anesthesiology (ESA) members [62]. Standardized fluid therapy is sparsely practiced in the US with less than 6% of ASA respondents having a facility-based written protocol while ESA members were five times more likely to have one.

Lack of standard criteria for fluid therapy results in significant clinical variability relative to the type and volume of fluid administered. This variability is linked to variable outcomes and makes it difficult to assess the effectiveness of different approaches [21,63]. A universal formula for an effective fluid management is fraught with difficulty because responses to fluid therapy vary widely between patients and not all patients benefit from fluids [64]. The complexity and individual variability of human physiology, presurgical morbidities, and the impact of different surgical procedures makes it easy to understand why a general, one-size-fits-all formula for fluid administration is unlikely to provide benefit.

### Methods

Based on the abovementioned acknowledgements, a group of 72 researchers, well known within the field of fluid resuscitation, were invited, via email, to attend a meeting that was held in Chicago in 2011 to discuss perioperative fluid therapy. From the 72 invitees, 14 researchers representing 7 countries attended, and thus, the international Fluid Optimization Group (FOG) came into existence. These researches, working collaboratively, have reviewed the data from 162 different fluid resuscitation papers including both operative and intensive care unit populations.

### IRB

There was no human research involved with this manuscript.

## Results

This manuscript is the result of 3 years of evidence-based, discussions, analysis, and synthesis of the currently known risks and benefits of individual fluids and the best methods for administering them. The results of this review paper provide an overview of the components of an effective perioperative fluid administration plan and address both the physiologic principles and outcomes of fluid administration.

We present our evidence-based suggestions and individualized algorithms for a standardized approach to perioperative volume therapy for surgical patients. We propose specific recommendations for fluid administration which are organized into seven tenets as follows: 1) fluid responsiveness, dynamic indices, and the gray zone; 2) considerations of the composition of crystalloids and colloids; 3) evidence-based guidelines and individualized algorithms; 4) perioperative fluid plan; 5) goal-directed therapy; 6) the fluid challenge; and 7) maintenance fluids.

Recommendations are italicized.

## Discussion

### Recommendations

#### Fluid responsiveness, dynamic indices, and the gray zone

In patients who have cardiac rhythms with regular *R*-*R* intervals and who are receiving controlled mechanical ventilation with tidal volumes between 8 and 10 ml/kg, fluid responsiveness is most effectively assessed using dynamic indices. These should be measured in a uniform manner before and promptly after each fluid intervention [65-69]. Currently used dynamic indices include systolic pressure variation (SPV), pulse pressure variation (PPV), stroke volume variation (SVV), and plethysmographic waveform variation (PWV). The clinical utility of dynamic parameters is limited by many confounding factors that must be clearly understood by the clinician utilizing them [70].

The role of echocardiography, both transthoracic and transesophageal, can be critical when evaluating both fluid responsiveness and cardiac function. In addition, echocardiography is of particular use when assessing volume responsiveness in patients undergoing open chest surgery where the predictive ability of dynamic indices is also reduced [71].

Static parameters (for example, right or left ventricular diastolic diameter) derived from transesophagic echocardiography (TEE) monitoring are not useful in predicting volume responsiveness [72]. On the other hand, echo-derived dynamic indices such as *delta* IVC and *delta* SVC diameter during positive pressure ventilation have shown to be effective for evaluating fluid responsiveness [73]. As with all echocardiographic techniques, image acquisition and interpretation requires considerable education and experience. Furthermore, equipment expense still remains a considerable barrier to widespread implementation.

While dynamic indices are excellent for predicting volume responsiveness, measured changes in cardiac output (∆CO) or stroke volume (∆SV) may be required to assure the effectiveness of a fluid bolus [74,75]. Dynamic indices can be used to predict when fluid therapy could be administered and when its administration should be stopped. Bolus volume therapy should be discontinued when a patient reaches that point on their Frank-Starling curve where further volume therapy will not augment cardiac stroke volume (dynamic index < 10%, ∆SV or ∆CO < 10%).

Dynamic indices have been repeatedly shown to accurately reflect fluid responsiveness and do so better than commonly used static hemodynamic parameters. These parameters have been validated and used to guide fluid therapy in a variety of surgical patients, including those undergoing major abdominal [42,50,68,76-78], cardiac [69,79-86], neurosurgical [87,88], and vascular surgery [89]. Static measures such as central venous pressure (CVP) may be invaluable during patient care [90]; however, CVP is not useful as a predictor of volume responsiveness.

Dynamic parameters should be an integral part of GDT protocols for those patients in which they can be accurately measured. ∆CO or ∆SV can be used in the remaining patients. Not taking into account the status of fluid responsiveness when making fluid therapy decisions is bound to result in unjustified fluid administration even when GDT is being used. In addition, dynamic parameters may precede continuously measured CO, heart rate, and blood pressure in alerting to the development of hypovolemia and may therefore trigger an early and justified fluid administration [91,92]. It is important to realize, however, that the presence of fluid responsiveness is not an absolute indication to give fluids. The decision to administer fluid therapy must be supported by proof of volume responsiveness, the need for hemodynamic improvement, and the lack of associated risk [93]. Fluid load *per se* is not always the correct therapy for hemodynamic instability.

The predictive ability of various dynamic indices has been compared in a number of studies. PPV had been found to be somewhat more accurate than the SPV and SVV [79,94,95]. However, it is difficult to determine a single cutoff point to predict fluid responsiveness. Cannesson *et al*. showed that, despite strong predictive value, there is a range of PPV values, named the gray zone (between 9% and 13%), for which fluid responsiveness cannot be reliably predicted in 25% of patients during general anesthesia [93]. Moreover, the gray zone limits may change according to the fluid management strategy to be applied [93]. Thus, when PPV enters the gray zone, uncertainty exists and clinicians should utilize other tools to assess fluid responsiveness. Furthermore, the range applied to PPV may not be applicable when SVV or other dynamic indices are used for determining volume responsiveness. The gray zone for each dynamic index requires its own definition [96].

The interaction between PPV and SVV (PPV/SVV) has also been studied as a measure of dynamic vascular compliance [97,98]. These combined parameters may be used to identify those hypotensive patients who have an underlying vasodilatory component to their hypotensive state and, thus, the need for vasopressor therapy [99].

Since pulse oximetry is a standard noninvasive intraoperative monitor, the respiratory variation in the plethysmographic waveform (PWV) is potentially the most commonly available dynamic parameter in mechanically ventilated anesthetized patients [100]. The major problem with the clinical use of PWV is the significant impact of vasoconstriction (for example, hypotension, hypothermia) on the plethysmographic waveform. However, an increase in the PWV may be the first sign of the development of a still-occult hypovolemia and should prompt the anesthesiologist to consider the immediate administration of fluids.

##### Limitations of dynamic indices

Fluid responsiveness measures cannot be used in all patients and at all times in many patients. Dynamic indices have a high predictive value in determining fluid responsiveness; however, specific criteria must be met in order to use these indices to assess fluid responsiveness. Intraoperative motion, electrosurgical equipment, and physiologic artifact (noise) can interfere with the accurate interpretation of dynamic indices. Four primary limitations may exist in the use of dynamic indices. First, arrhythmias (for example, atrial fibrillation) preclude the use of SPV, PPV, SVV, and PWV to predict volume responsiveness, while inferior and superior vena cava variability remain accurate. The same limitation of SPV, PPV, SVV, and PWV is seen in subjects having varying levels of spontaneous inspiratory efforts. Again, inferior vena and superior vena cava diameter variability may remain predictive of volume responsiveness during spontaneous breathing. Second, if tidal volumes are <8 ml/kg, then the negative predictive value of SPV, PPV, SVV, and PWV is decreased whereas threshold values >13% variation still retain their positive predictive value. Third, marked decreases in chest wall compliance will decrease the positive predictive value of all indices whereas intra-abdominal hypertension may mask hypovolemia but will not alter the volume responsiveness prediction value of these indices. Fourth, in the setting of acute cor pulmonale, with marked ventricular interdependence, one will see a paradoxical positive SPV, PPV, SVV, or PWV which will increase more with fluid resuscitation. Thus, when dynamic indices are utilized for guiding fluid therapy, some measure of the effectiveness of augmented perfusion should be considered.

Importantly, if these indices have values >20%, then the subject is clearly volume responsive. However, values from 9% to 13% may represent a ‘gray zone’ with less positive and negative predictive values and greater patient-to-patient variability. In these cases and when any of the above limitations precludes the use of these parameters, one may consider performing a fluid challenge or passive leg raising (PLR) maneuver [101]. In contrast to a mechanical breath that normally reduces CO, the PLR causes an ‘endogenous fluid challenge’ which will increase CO in ‘responders’. The PLR maneuver with a sensitivity of 89.4% and a specificity of 91.4% for predicting volume responsiveness is best coupled with minimally invasive cardiac output monitors that can track changes in stroke volume and cardiac output dynamically and in real time regardless of the mode of ventilation [102,103]. The execution of PLR, however, necessitates a major positional change, which generally makes it impractical for intraoperative use. However, there are instances in the operating room (OR) where postural changes may induce a hemodynamic response that may serve as a diagnostic maneuver of fluid responsiveness.

*We recommend that dynamic parameters be used as an integral part of GDT protocols. The limitations of each dynamic index must be taken into consideration as well as the concept of a gray zone. Dynamic parameters neither provide a measure of fluid bolus effectiveness nor should they to be used as an indication to give fluids. The final decision to administer fluids must be supported by the apparent need for hemodynamic improvement, the presence of fluid responsiveness, and the lack of associated risk.*

#### Composition of fluid therapy: crystalloids and colloids

There has been extensive research evaluating the risks and benefits of specific types of fluids and developing alternative solutions that restore effective circulatory volume and enhance microcirculatory flow. Despite all these efforts, the ideal resuscitation fluid or combination of fluids remains undefined.

There are three fluid categories - crystalloids, colloids, and blood. Each has its unique characteristics and role in fluid therapy. This discussion will focus on crystalloid and colloid therapy.

- *Crystalloids* are electrolyte solutions which are best used to replace extracellular volume losses from perspiration, respiration, and urine output. Although crystalloids increase vascular volume and may improve hemodynamics, the effectiveness is transient and less than colloid solutions. Crystalloids can be classified by their composition and osmolality. Normal saline (NS) is slightly hypertonic at 308 mOsm/l, and lactated Ringer’s (LR) is slightly hypotonic at 273 mOsm/l comparing to plasma osmolality. Plasmalyte is the most balanced isotonic electrolyte solution and has an osmolality of 294 mOsm (Table 2).

**Table 2:** **Commonly applied crystalloid solutions: osmolality, cationic, and anionic composition**

**Fluid**	**Osmolality (mOsm/l)**	**pH**	**Na** ^**+**^ **(mEq/l)**	**K** ^**+**^ **(mEq/l)**	**Ca** ^**++**^ **(mEql/l)**	**Lactate (mEql/l)**	**Cl** ^**−**^ **(mEq/l)**	**Acetate (mEq/l)**
Plasma	285 to 295	7.4	142	4	5	27	1	
0.9% saline	308	5.5	154				154	
Lactate Ringer’s	273	6.5	130	5.4	2.7	29	109	
Plasmalyte	294	7.4	140	5			98	27
- *Colloids* are solutions of macromolecular solutes that exert a colloid osmotic pressure across the microvascular tissue barrier and retain fluid in the intravascular bed. Colloids efficiently increase vascular volume, preload, cardiac output, and tissue perfusion in volume responsive patients. Many of the GDT trials that have shown improved outcomes employ the use of iterative infusions (small volume boluses) of colloid (Table 1) [23,29-59]. Compared with the hemodynamic and volume-restoring effects of crystalloid therapy, equi-efficacious volumes of colloid are smaller; thus, colloid use may be considered an approach to limiting total volumes, which may contribute to better outcomes.

Comparison with the plasma composition. Commonly used intravenous fluids vary considerably in osmolality, ionic composition, and pH. Crystalloid selection should be based upon individual patient need with clinical consideration of these components.

The choice of fluids is largely based on traditional beliefs, context of practice, location [104], and cost. For example, in comparing the use of colloid to crystalloid for treating hypovolemia, clinicians from the UK, China, and Australia rely primarily on colloid therapy (55% to 75% of time), whereas only 13% of clinicians in the US use colloid for treating hypovolemia [105].

Results of clinical trials comparing fluid resuscitation with colloids and crystalloids in different populations have been conflicting. Most recently, as highlighted in clinical trials and meta-analyses, the safety of using specific colloids (starches) for fluid resuscitation has been questioned [106,107]. Table 3 shows the main current concerns regarding specific crystalloids and colloids [108-114].

**Table 3:** **Main current concerns regarding the use of specific crystalloids and colloids**

**Solution**	**Concerns**	**Literature**
Normal saline	Hyperchloremic acidosis	Hyperchloremia after noncardiac surgery is independently associated with morbidity and mortality [108]
	Reduction of renal perfusion	May contribute to acute renal injury [109,110]
Starch solutions	Acute kidney injury and increased requirement of renal replacement therapy	Critically ill septic patients [111-114]
	Increased mortality	Critically ill septic patients [112,114]
	Increased need for PRBC transfusion	Critically ill septic patients [114]

Results of clinical trials comparing fluid resuscitation with colloids and crystalloids in different populations have been conflicting. This table summarizes current concerns regarding specific crystalloids and colloids.

The CRISTAL trial compared the effects of fluid resuscitation with colloids *versus* crystalloids on mortality in patients admitted to the ICU with hypovolemic shock. There was no difference in 28-day mortality between patients resuscitated with crystalloids or colloids. However, the 90-day mortality was significantly reduced in patients treated with colloids [115]. On the other hand, in patients with severe sepsis and capillary leakage, the fluid-sparing effect of colloids appears to be smaller than anticipated [112,113]. However, balancing the CRISTAL trial, the recently completed ALBIOS trial comparing 20% albumin and crystalloid *versus* crystalloid in 1,818 septic patients demonstrated that the colloid group had a higher mean arterial pressure during the first 7 days whereas there were no differences in the total amount of fluids administered between the two groups and both 28-day and 90-day mortality rates were similar. Thus, there is no compelling evidence that adding colloids to fluid resuscitation materially alters clinically relevant outcomes [116].

Given the evidence of harm and lack of significant clinical benefit in critically ill patients, when considering the administration of synthetic colloids, the anesthesiologist should first assess patient-specific risk. There is no evidence that the deleterious effects of starch-based colloids occur with albumin. The beneficial hemodynamic effects of colloid in GDT groups *versus* standard of care therapy suggest benefit of nonstarch colloids such as albumin. It should be noted that the deleterious effects of starches have largely been reported in ICU trials where starch therapy was used for multiple days. In contrast, the beneficial effects of perioperative GDT trials that included starch-based volume therapy were only of limited duration and thus exposure. For this reason, we cannot conclude that the deleterious effects of starches shown in the ICU population are generalizable to the limited use that occurs in the immediate surgical space. Serious thought to the individual surgical patient’s co-morbidities, especially acute kidney injury, can inform the anesthesiologist of potential increased risk of starch-based colloid therapy.

A recent Cochrane meta-analyses has concluded, however, that there is no evidence from randomized clinical trials that resuscitation with colloids, instead of crystalloids, reduces the risk of death in patients with trauma, burns, or following surgery [117]. Common to all meta-analyses and systematic reviews, inclusion of studies whose interventions and patient characteristics are often insufficiently comparable and, therefore, the calculation of a summary effect measure may be questioned. The resuscitation regimen, the type of colloid or crystalloid, and the end points that guided resuscitation differed between trials. Further, the value of colloids when used as part of GDT may be apparent only in high-risk surgery patients.

Similar caution and approach should be applied to other synthetic colloids such as dextran and gelatin. Scant clinical evidence exists as to either benefit or harm regarding to the administration of other colloid solutions such as dextran or gelatin to surgical patients. Siting theoretical safety concerns, some authors posit caution for the routine use of these fluids in surgical patients [117]. It should not be assumed that results from fluid resuscitation trials in ICU populations apply to surgical patients. Properly powered, prospective trials comparing different fluids in defined patient populations undergoing specific surgical procedures are needed [118].

*We recommend crystalloid solutions for routine surgery of short duration. However, in major surgery, the use of a goal-directed fluid regimen containing colloid and balanced-salt solutions is recommended. Though a black box warning for the use of starch solutions exists within the US, there is limited data relative to their harm in the perioperative space. Careful consideration should occur in patients with known renal dysfunction and/or sepsis prior to administering starch solutions.*

#### Evidence-based guidelines and individualized algorithms

Disagreement about optimal perioperative fluid therapy is exacerbated by the lack of uniform definitions for standard, restrictive, and supplemental fluid delivery [21]. This, in turn, hinders comparisons of published studies [119]. Better definitions of the fluid regimen will help facility champions to develop local guidelines and algorithms.

*Guidelines* are general suggestions of care based on *principles* extracted from *evidence-based* findings and consensus. *Algorithms* are highly specific as to the variable(s) used, their target values, and their specific steps.

The difference between a guideline and an algorithm is important. Guidelines do not provide sufficient detail to reduce variation of care. Two anesthesiologists could strictly adhere to a guideline, but their specific fluid therapy delivered to an identical patient could be quite different. Even one of these individual anesthesiologist’s practices for two identical patients could differ from 1 day to another. Improved outcomes, reduced readmissions, and reduced costs have resulted when quality improvement programs have been implemented to reduce variability [120,121]. The implementation of algorithms or detailed protocols into routine anesthetic care is far more important than adherence to guidelines when attempting to reduce clinical variation. Enhanced recovery after surgery (ERAS) protocols represent multidisciplinary perioperative care pathways that seem to be associated with significant reductions of the surgical stress response, complications, and hospital length of stay (HLOS) [122,123]. A recent clinical trial showed that the implementation of an ERAS protocol for colorectal surgery at a tertiary medical center was associated with a significant reduction of HLOS for both open and laparoscopic colorectal surgeries. The authors, however, were not able to show significant difference in the total medical costs for patients in the ERAS pathway *versus* the traditional care group [124]. Importantly, fluid therapy was only one of the 23 steps that were implemented within the study protocol. Unfortunately, we are unable to define the impact of this critical step as the study was not designed to do so. It is likely that some interventions are more important than others relative to reducing complications, readmissions, and total hospital costs, and some may be nonessential. Indeed, Loftus *et al*. demonstrated that significant reductions in complications and readmissions could be realized with the implementation of a simple two-step ERAS protocol focusing on early ambulation and alimentation following colorectal surgery [121].

**Figure 2:**
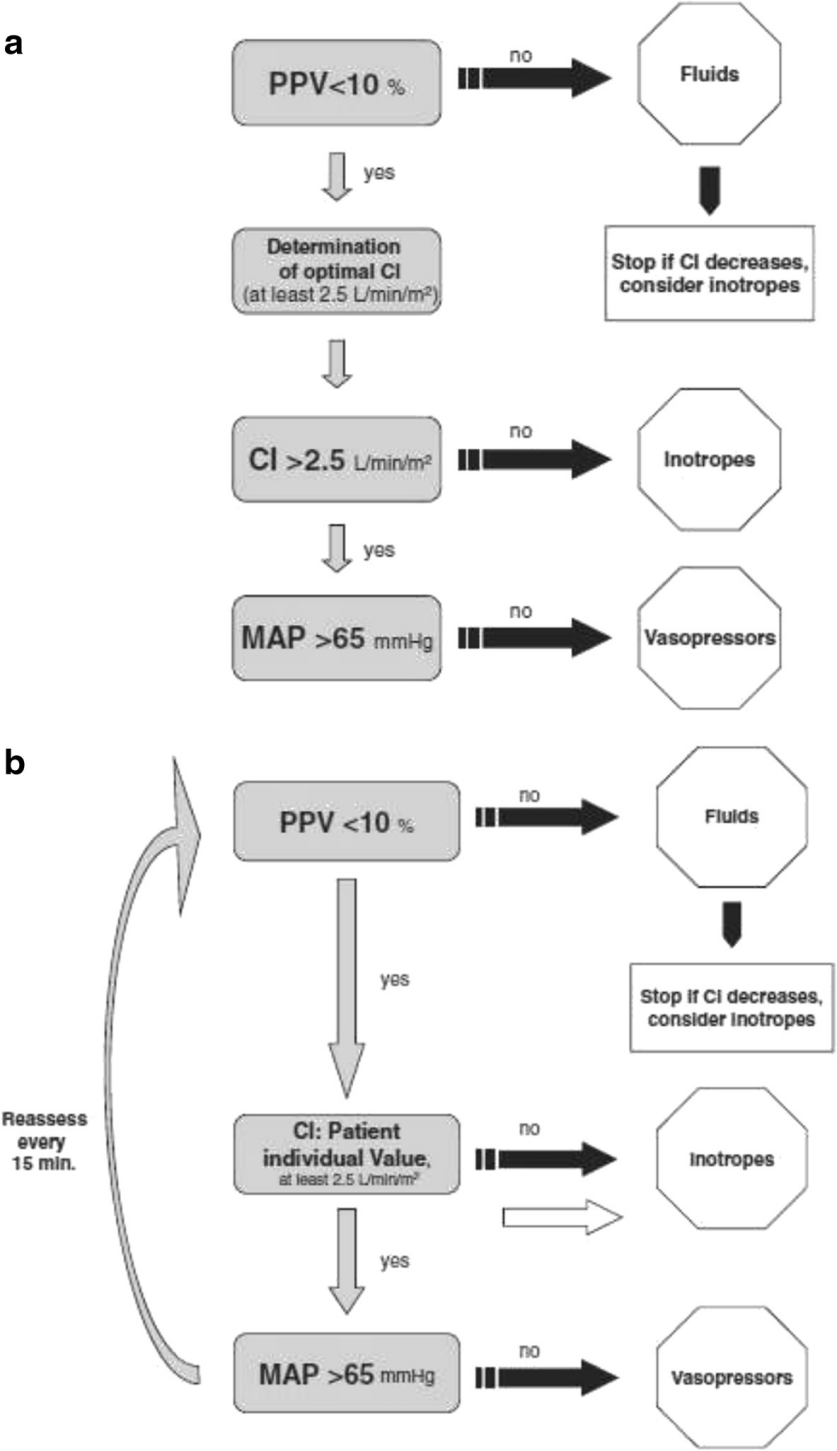
**Goal-directed hemodynamic algorithm to guide intraoperative volume therapy in major abdominal surgeries: (a) initial assessment and treatment and (b) further intraoperative optimization [** 58 **] (used per BioMed Central’s creative commons license).** PPV, pulse pressure variation; CI, cardiac index; MAP, mean arterial pressure.

Importantly, algorithms should not be fixed, they should allow for individualizing fluid therapy based on changing physiologic need and response to fluid and drug therapy. Algorithms can become very detailed and are likely to be best implemented with computerized decision support [125,126]. Both guidelines and algorithms can be displayed in flow chart format or computerized. Figure 2 provides an example of fluid therapy algorithm [58]. There will be many perioperative events that require deviation from algorithms. There is no substitute for medical training and expertise; however, deviation from any protocol should have a rational basis. Furthermore, nonadherence is often an opportunity to better understand and improve guidelines and algorithms.

Computerized decision support and implementation of closed-loop fluid administration has been described. Significant regulatory challenges exist before these systems can be introduced into clinical practice [127,128]. Recently, the first clinical use of a closed-loop fluid management system was reported [129]. With this approach, 91% of the physiologic targets were obtained. The authors suggest that the use of a closed-loop fluid management system may ease the implementation of algorithms, increase compliance with best practices, and relieve clinicians from time-intensive repetitive tasks [128].

*We recommend the use of algorithms as part of the perioperative fluid plan. These should be available and easily accessible within all operating rooms. We encourage continued development, refinement, and testing of computerized decision support tools.*

#### The perioperative fluid plan

**Figure 3:**
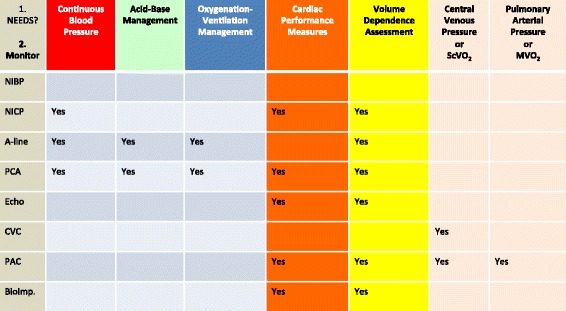
**A rational approach to intraoperative monitoring.** A useful approach for assessing the needed level of hemodynamic monitoring based on the patient status, surgical risk, and clinical management requirements (*what are my management needs?*)*.* NIBP, noninvasive blood pressure; ECG, electrocardiogram; A-line, arterial catheterization; NICP, noninvasive continuous pressure; CVC, central venous catheter; ECHO, transthoracic or transesophageal echocardiography; PAC, pulmonary artery catheter; ScVO_2_, central venous oxygen saturation; MVO_2_, mixed venous oxygen saturation; PCA, pulse contour analysis; BioImp, bioimpedance or bioreactance.

The use of protocols for perioperative hemodynamic support which enhance tissue perfusion has been shown in multiple meta-analyses to reduce organ dysfunction, mortality, and HLOS [7,10,130,131]. These outcomes are especially evident when applied to the sickest patients [132]. A fundamental aspect in any perioperative protocol is the use of a fluid therapy plan that should be centered on physiological principles, evidence-based medicine, and local expertise. Given the absence of an internationally accepted fluid protocol or comprehensive fluid therapy, guidelines creating local standards become imperative. The anesthesiologist should have an individualized perioperative fluid optimization and hemodynamic monitoring plan for each surgical patient based upon the following:

1. Patient status (health, age, physiology, and co-morbidities);
2. Surgical risk (procedure, approach, and surgical expertise);
3. Selection of hemodynamic monitoring based upon patient and surgical risk as well as the anesthesiologists’ clinical management needs (continuous blood pressure, cardiac performance, volume responsiveness, acid-base management, optimize oxygenation and ventilation, central venous and/or pulmonary artery pressures, central or mixed venous oxygenation). Figure 3 shows a rational approach to intraoperative monitoring.

##### Hemodynamic monitoring

Vincent *et al*. [133] proposed key principles regarding hemodynamic monitoring. Some of these principles are summarized in Table 4. In summary, the best choice for monitored variables depends on the type of patient, the question being asked, and the condition being managed or anticipated (Figure 3). It is crucial to understand that it is not the monitoring itself that can improve outcomes, but the changes in therapy guided by the data obtained [133,134]. Advantages of noninvasive or minimally invasive approaches are obvious. Further considerations of specific monitors are beyond the scope of this review.

**Table 4:** **Principles of hemodynamic monitoring (Vincent** ***et al*** **.)** [133 **]**

**Principle**	**Rational**
No hemodynamic monitoring technique can improve outcome by itself	If the data are interpreted or applied incorrectly the resultant change in management will not improve patient outcome and may be deleterious
Monitoring requirements may vary over time	Optimal monitoring system depend on the individual patient, the problem already present or potentially arising, and the devices and expertise available. Different monitoring techniques can sometimes be used to complement each other.
There are no optimal hemodynamic values or targets that are applicable to all patients	Targets and alarms should thus be individualized and reassessed regularly
Any variable on its own provides just one piece of a large puzzle	Variables should be combined and integrated
Continuous measurements of hemodynamic variables is preferable	Real time information and trends are useful on the perioperative settings

This table highlights a fundamental truth regarding hemodynamic monitoring and patient outcomes: Hemodynamic monitoring devices do not change patient outcomes unless coupled to treatments or treatment protocols which are known to improve outcome.

In low-risk patients and low-risk surgery, the use of ASA standard monitors is often sufficient. However, if the associated risk or surgical procedure escalates, or if unexpected patient instability develops, additional expertise and/or monitoring are requisite. The advancement of monitoring is not without increased risk and cost; thus, these tools should only be applied when needed to provide a means to better detect and treat tissue mal-perfusion and potential organ dysfunction. Fluid therapy is a cornerstone for perioperative medicine, but clarity on when not to infuse fluids is as important as when to infuse. Hemodynamic and other advanced monitoring is often the best means to assess and assure the optimization of intravascular volume, pressure, perfusion, and oxygenation.

Invasive cardiovascular monitors can be considered in patients in whom tight hemodynamic control is needed to prevent rapid organ deterioration, for example, significant heart or brain disease and in high-risk surgical cases, for example, aortic and heart surgery. Indices from commonly used invasive monitors include intra-arterial pressure from an arterial catheter, right and left heart filling pressures, and central or mixed venous oxygenation saturations from central venous or pulmonary artery catheters, respectively.

There are several commercially available noninvasive or minimally invasive technologies that employ arterial pulse contour analysis, bioimpedance, or bioreactance and which provide continuous cardiac output, dynamic indices, and systemic vascular resistance. Finally, echocardiography, transthoracic and transesophageal (TEE), is becoming increasingly utilized in at risk patients. Mastering cardiac performance and volume assessment by TEE may improve currently available GDT algorithms.

*We recommend that a perioperative fluid plan be developed by each department, facility, or health system and used by all anesthesiologists. Clinical needs, invasiveness, accuracy, and precision of available technologies should be considered when selecting monitoring devices.*

#### Goal-directed therapy

Until recently, perioperative fluid replacement was guided primarily by estimates of known or anticipated fluid deficits and replacing these using fixed calculations for administration of intravenous fluid. Little outcome data exist that support the widespread use of fixed perioperative fluid regimens. Recent approaches have focused attention on the type of surgery being performed and the impact of the following outcomes: 1) the type of fluid being administered; 2) the timing of fluid administration; 3) the rate of fluid administration [135,136]; 4) the total amount of fluid administered; and 5) the best measures to both optimize and individualize perioperative fluid therapy [64].

Effective fluid therapy algorithms incorporate GDT. Table 1 lists trials of GDT for specific surgical procedures and the target goals and algorithms employed and their impact on outcomes. Algorithms should incorporate strategies and clinical pathways for patients who do not respond to fluid therapy (‘nonresponders’) and co-morbidities.

There are two overall challenges for fluid optimization as follows: 1) how to best identify hypovolemia and tissue hypoperfusion; and 2) how to best optimize vascular volume, cardiac filling, global, and regional perfusion and tissue oxygenation.

##### Identifying the need for hemodynamic support

The most common parameters that are used to guide the need for hemodynamic support and perioperative fluids include clinical experience, urinary output, mean arterial pressure, and CVP [62,137]. Other variables that may be available include cardiac output, systemic vascular resistance, serum lactate, and central or mixed venous oxygen saturation. Peripheral pulses, skin temperature, appearance, and turgor are subjective measures that require significant clinical experience and acumen to be used effectively. The response of the CO to fluid administration depends on the preload status and on the contractile state of the heart, namely the slope of both the RV and LV function curve [138]. This explains why some hemodynamic variables, for example, central venous pressure, can fail to predict the response of the CO to fluid administration [65,79,139-141]. Only half (!) of critically ill and high-risk surgical patients, in whom fluid loading seems to be indicated, do indeed increase their CO in response to fluid loading (‘responders’), while the other half (‘nonresponders’) can be loaded with fluids unnecessarily [72]. When making a decision about fluid administration, it is best to rely on the assessment of *fluid responsiveness*, that is, a measure of the change in CO in response to an increase in preload [142] as discussed below. The recently completed OPTIMISE trial when coupled with a meta-analysis of prior clinical trials demonstrated that using cardiac output targets to guide intraoperative fluid resuscitation decrease postoperative complications and reduce hospital length of stay [59].

##### Controversies within the GDT literature

Although the goal-directed fluid therapy concept was first suggested more than 30 years ago [143], there remains no consensus about the most effective goals for fluid therapy or the most appropriated monitoring methods. As such, despite evidences demonstrating potential benefit of this technique in several disease states [144], GDT remains a well-accepted concept that has not yet translated to an established standard of care [145]. As exemplified in Table 1, directed comparison between studies is hampered by the large range of goals and methods for monitoring the inconsistency of study designs and the lack of common control groups [145]. Accordingly, there is an urgent need to address this research gap, providing high-quality evidence in support to different goals and methods of monitoring fluid therapy.

*While the benefits of perioperative goal-directed fluid therapy have yet to be proven, the bulk of clinical research supports the implementation of a two-step GDT plan which is to begin immediately after induction of anesthesia. First, determine if the patient requires hemodynamic support or augmentation of cardiovascular function. Second, if the need is apparent and the patient is fluid responsive, fluid bolus therapy should be considered and guided by continual, and if available continuous, assessment of fluid responsiveness as described below.*

#### The fluid challenge

A fluid challenge is one of the best tools that the anesthesiologist has for assessing fluid responsiveness. To test fluid responsiveness, a change in preload (fluid bolus) must be induced while monitoring the subsequent change in stroke volume, cardiac output, and dynamic indices [146].

The use of a fluid bolus provides two advantages as follows:

1. a means to assess the patient’s response to fluid with changes in dynamic indices and static indices of volume, flow, and oxygenation;
2. a prompt increase in intravascular volume and usually a needed improvement in flow (cardiac output).

A fluid bolus is a provocative test of the circulation, similar to the use of a step function in engineering to define a system. The use of a ‘test’ that uses a small amount of fluid (bolus) to assess the volume responsiveness may reduce the risk of a too liberal fluid strategy and the possible consequences of fluid overload. These tools help to determine the requirements for additional fluid therapy avoiding the deleterious consequences of fluid overload through its small volume and targeted administration [147].

It is important to stress that the fluid challenge technique is a test of the cardiovascular system. It allows clinicians to assess whether a patient has enough preload reserve to increase stroke volume with further fluids. Fluid therapy should be considered after a positive response to a fluid challenge. In contrast to a single fluid challenge, fluids can also be infused in a controlled fashion based on an algorithm by repeating the fluid challenge as long as there is a positive response. This controlled approach is called stroke volume maximization and is the cornerstone of most goal-directed therapy protocols [38]. Thus, the only reason to perform a fluid challenge is to increase a patient’s stroke volume; if this does not happen, further fluid administration is likely to be harmful [148].

A fluid challenge should comprise four separate orders: the type of fluid to be infused, the volume of fluid to be infused, the rate of the infusion, and the stopping rules if untoward effects are seen before the full amount of the bolus is infused. For rapid infusions of very small boluses of fluid (for example, 250 ml crystalloid over 1 to 2 min), stopping rules are probably not necessary. But if larger amounts of fluids or longer infusion times are used, clear stopping rules are important to prevent right heart failure or pulmonary edema.

Although no consensus is available for the type and exact dosing of fluid administration, boluses are best delivered at a rapid rate (5 to 10 min) with prompt assessment of the physiologic response. The magnitude of this response helps to determine the effectiveness of the fluid challenge as well as the requirements for additional fluid therapy. Taken together, this approach avoids the deleterious consequences of fluid overload [147]. The peak and sustainment of improvement in dynamic and static variables after a fluid bolus is dependent on both the physiologic state and fluid composition. Moreover, sustainment of the response after bolus can be reduced in the presence of continuing hemorrhage.

Establishing volume status is complex, making accurate prediction of an increase in stroke volume upon fluid load challenging. However, under conditions of hypovolemia and inadequate perfusion, there is greater vascular retention of infused volume due to physiologic compensatory mechanisms that act to maintain normal volume, pressure, and perfusion. These compensatory mechanisms include the renal response to elevated vasopressin, angiotensin, and aldosterone; reduced capillary filtration due to reduced venous and capillary pressures; and decreased capillary hydraulic conductivity due to fluid composition and decreased levels of atrial natriuretic peptide (ANP) [149,150]. The use of a limited selection of specific volumes and delivered at set rate(s) of infusion provides a standardized test for volume responsiveness and a better means for the comparative assessment of changes in volume responsiveness.

*We recommend bolus therapy rather than continuous infusion when the goal is to improve pressure, perfusion, and oxygen delivery. Standardization of the fluid bolus relative to fluid composition, volume, infusion rate, and time to post bolus assessment should be implemented. The variables used for assessing the effectiveness of the fluid bolus should include appropriate changes in cardiac output or stroke volume.*

#### Maintenance fluids

Traditional perioperative fluid administration is guided by estimates of both the preoperative fluid deficit and by ongoing sensible and insensible intraoperative fluid losses. The notion that all surgical patients are hypovolemic due to prolonged fasting, bowel preparation, and ongoing losses from perspiration and urinary output is unfounded. Preoperative volume status is typically unknown and should not be presumed to be either adequate or inadequate. Blood volume varies considerably between patients depending on gender, weight, and oxygen consumption [151-153]. Moreover, effective circulatory volume varies when patients are under anesthesia [154]. Furthermore, our understanding of fluid shifting has changed and the so-called ‘third space’ has mostly been abandoned [155]. Additionally, perioperative deficits and insensible losses are often overestimated. Almost 40 years ago, direct measurements of basal evaporation rate from skin, airway and large exposure of bowel showed that fluid loss is 0.5 to 1.0 ml/kg/h during major abdominal surgery [156]. Despite this fact, many current textbooks and guidelines for perioperative fluid management in major abdominal surgery suggest large amounts of crystalloids (5 to 7 ml/kg/h) for maintenance of intraoperative circulating volume [6].

The majority of the patients present with a minor functional intravascular deficit before surgery (200 to 600 ml) that is unlikely to have clinical significance [7]. This may explain why prophylactic fluid boluses have no major effects on the incidence or severity of anesthesia-related hypotension [157]. Research has shown that fasting from solid food for 6 h and fluids for 2 h prior to surgery is safe and improves outcomes compared with longer fasting periods [122]. Moreover, mechanical bowel preparation before elective abdominal surgery has been strongly challenged. Indeed, current ERAS guidelines discourage bowel preparation routinely for colonic surgery [122].

In the clinical context of ambulatory surgery in low-risk patients, a more liberal fluid strategy may be beneficial. Up to 20 to 30 ml/kg/h of crystalloid infusion reduces postoperative dizziness, drowsiness, pain, nausea, vomiting, and hospital length of stay [158-160]. To the contrary, studies of patients undergoing major surgery may favor a more restrictive fluid regime [17,24], particularly in lengthy surgical procedures (>6 h) where fluid overloading significantly increases interstitial edema [161]. Because microvascular permeability peaks at 3 to 4 h after surgical injury [162], lengthy procedures are thus associated with capillary leakage and enhanced edema formation.

*We recommend that maintenance fluids be administered at a rate of 1 to 2 ml/kg/h for patients undergoing procedures of longer duration or magnitude. Patients undergoing outpatient procedures may benefit from higher maintenance fluid rates.*

## Conclusions

Although perioperative fluid management remains a highly debated subject, data suggests that goal-directed fluid therapy with the objective of hemodynamic optimization can reduce complications after major surgery. Specific hemodynamic goals include maintaining adequate circulating volume, perfusion pressure, and oxygen delivery. Lack of standard criteria for perioperative fluid therapy results in significant clinical variability relative to its administration.

In summary, fluids should be treated as any other intravenous drug therapy, and thus, careful consideration of its timing and dose is mandatory. A perioperative fluid plan should be developed which is easily understood and used by all anesthesiologists within a group, facility, or healthcare system. Determining both the need for augmented perfusion and fluid responsiveness is fundamental when making fluid therapy decisions to avoid unjustified fluid administration. Balanced crystalloid solutions should be given for short duration/low-risk surgical patients. Procedures of higher complexity are best managed with a combination of crystalloid and colloid therapy. When considering the administration of starch containing solutions, the anesthesiologist should first assess patient-specific risk. Finally, we recommend the use of algorithms as part of the perioperative fluid plan.

## Abbreviations

ASA: American Society of Anesthesiologists
CI: cardiac index
CO: cardiac output
CVC: central venous catheter
CVP: central venous pressure
DO_2_: oxygen delivery
ECHO: transthoracic or transesophageal echocardiography
ERAS: enhanced recovery after surgery
FFP: fresh frozen plasma
FOG: Fluid Optimization Group
FTc: corrected flow time
GDT: goal-directed therapy
GEDVI: global end-diastolic volume index
HLOS: hospital length of stay
HR: heart rate
ICU: intensive care unit
ITBVI: intrathoracic blood volume index
IVC: inferior venous cava
LR: lactate Ringer’s
LV: left ventricle
MAP: mean arterial pressure
MVO_2_: myocardial oxygen consumption
NS: normal saline
O_2_ER: oxygen extraction rate
PAC: pulmonary artery catheter
PCA: pulse contour analysis
PLR: passive leg raising
PONV: postoperative nausea and vomit
PP: pulse pressure
PPV: pulse pressure variation
PRBC: packed red blood cells
PVI: pulse variability index
PWV: plethysmographic waveform variation
RV: right ventricle
ScvO_2_: central venous oxygen saturation
SPV: systolic pressure variation
SV: stroke volume
SVC: superior venous cava
SVI: stroke volume index
SVV: stroke volume variation
TEE: transesophagic echocardiography
UO: urinary output
